# Enhancing 3D Printability of Black Soldier Fly Protein-Based Composite Gels by Incorporating Grape Seed Anthocyanin: Rheology, Water State, Protein Secondary Structure, and Microstructure

**DOI:** 10.3390/ma19143005

**Published:** 2026-07-12

**Authors:** Wenyue Deng, Jingjing Liao, Chaofan Guo

**Affiliations:** 1 School of Materials Science and Technology, Monash University, Melbourne 3800, Australia; wenyue_deng@163.com; 2 School of Materials Science and Technology, Central South University, Changsha 410083, China; 3 College of Landscape and Horticulture, Yunnan Agricultural University, Kunming 650201, China; jingjingliao@ynau.edu.cn; 4 School of Food Science and Technology, Kunming University of Science and Technology, Kunming 650500, China

**Keywords:** 3D printing, microstructure, rheology, black soldier fly protein gel, grape seed anthocyanin

## Abstract

**Highlights:**

- The incorporation of grape seed anthocyanin (GSA) significantly improved the 3D printing performance of the black soldier fly protein composite gels;
- GSA increased the deformation resistance of the composite gels and promoted the transition from disordered to ordered protein secondary structures;
- GSA promoted protein aggregation, thereby forming a new network that acted as a structural scaffold;
- Adding excessive GSA can have undesirable effects on 3D-printed black soldier fly protein (BSFP) composite gels;
- The BSFP composite gel containing 3% GSA exhibited the best 3D printing performance.

**Abstract:**

This study used black soldier fly protein (BSFP) as a base material and added 0%, 1%, 2%, 3%, 4%, and 5% of grape seed anthocyanidins (GSAs) to prepare composite gels. Through the combined use of low-field nuclear magnetic resonance, Fourier transform infrared spectroscopy, scanning electron microscopy, and rheometry, the relationships among GSA dosage (0–3%), gel structural properties (secondary protein conformation, water status, and microscopic morphology), and rheological printability were systematically evaluated. It was found that the better GSA content fell within 1–3%, and under this condition the extrusion-type 3D printing performance of the composite gels was significantly enhanced. At a 3% addition amount, the proportion of disordered conformations decreased (random coiling decreased from 15.93% to 15.46%), the ordered structure increased (β-sheet increased from 35.25% to 35.43%), and deformation resistance was enhanced. Low-field nuclear magnetic resonance showed an increase in the proportion of non-flowing water and an increase in physical constraints. Scanning electron microscopy showed a reduction in pore size and a thickening of pore walls, forming a denser 3D network. Rheologic analysis indicated that 3% GSA reached the maximum zero-shear viscosity (η_0_) and that the storage modulus (G′) and loss modulus (G″) were higher in the experimental group than those in the control group. Printing fidelity increased from 45.73% in the control group to 60.08% in the 1% group, 62.14% in the 2% group, and 71.05% in the 3% group (*p* < 0.05). The 3–5% groups (fidelity: 71.05–75.66%) all achieved hollow cylindrical printing without collapse and had excellent self-supporting performance. However, excessive addition (4–5%) caused excess GSA to adsorb onto the protein skeleton surface, reducing the apparent viscosity and damaging the printing performance. Based on all the indicators, the composite gel with 3% GSA achieved the best balance between printability and structural integrity. Our research offers a new idea for using flavonoid compounds to improve the 3D printing performance of insect protein gels. The prepared composite gels can be used as food printing inks and applied to personalized nutrition customization, functional food development, and sustainable protein alternative product fields.

## 1. Introduction

The world population is expected to reach 9.7 billion by 2050, requiring some 410 billion kilograms of protein each year to meet global demand [1]. Insects, as a renewable resource, have protein contents comparable to those of conventional protein sources, yet they remain underutilized [2,3]. Insects exhibit high feed conversion efficiency, efficient land utilization, remarkable reproductive capacity, short breeding cycles, and simple cultivation requirements, making them ideal candidates for protein provision [4,5].

The black soldier fly (*Hermetia illucens*) is a saprophagous insect belonging to the family Stratiomyidae in the order Diptera [6]. Owing to its rapid reproduction, large biomass, broad feeding habits, high absorption and conversion efficiency, ease of management, low rearing cost, and good palatability for animals, it has been widely utilized as a biological resource [7]. Black soldier fly biomass is particularly rich in protein, with the crude protein content of dried larvae ranging from 44% to 48%. This is comparable to plant-derived protein feeds such as broad beans and sunflower seeds, making it a typical high-protein biological resource [8]. It should be noted, however, that the widely cited range of 44–48% crude protein is typically obtained from defatted larval meals or larvae reared under optimized dietary regimes and thus does not represent an invariant benchmark. In the present study, the whole black soldier fly larval powder used for 3D printing contained 24.5% crude protein—a value lower than that reported in the literature range. This discrepancy primarily arises from the use of whole (non-defatted) powder, differences in rearing substrate composition, post-harvest drying conditions, and the nitrogen-to-protein conversion factor applied. Rather than diminishing the relevance of our work, this lower baseline provides a practically meaningful and economically realistic scenario for evaluating the effectiveness of anthocyanin-mediated gel modification, since whole-larval powders are more commonly handled in actual food processing chains. However, due to food neophobia, many consumers are reluctant to accept insect-based foods, including black soldier fly products. A subset of consumers has indicated a preference for insect-derived foods that more closely match their expected flavor and visual profiles. Studies have shown that when the distinctive appearance and odor of insects are not recognizable, consumer acceptance tends to increase. 3D food printing technology offers a promising solution to this challenge, namely processing insect proteins into products with customized shapes and attractive appearances, thereby masking the original appearance of the insects [9,10]. Research has shown that when insect food is processed into familiar or visually acceptable forms through technologies such as 3D printing, consumers are more willing to accept it [11,12].

With the advancement of science and technology, cutting-edge manufacturing techniques are increasingly integrated with conventional technologies, thus reshaping the conventional limits of food processing. 3D food printing constitutes an innovative digital manufacturing method that enables layer-by-layer construction of edible products. It combines additive manufacturing with food ingredient processing. Via layer-by-layer assembly of edible materials based on a digital template, novel food structures can be fabricated [13,14,15,16]. Food 3D printing can meet the specific demands of different consumer groups in terms of nutrition, texture, color, and shape [17,18,19]. In recent years, this technology has attracted substantial interest from researchers [20]. At present, the repertoire of food 3D printing comprises four principal techniques—extrusion-based printing, inkjet printing, selective laser sintering, and binder jetting. Extrusion-based printing constitutes the most widely utilized of these [21]. This technique necessitates printing materials with proper rheological and mechanical behavior, making them capable of easy nozzle flow coupled with reliable dimensional stability upon layer deposition [22,23,24]. Nonetheless, the variety of edible substrates compatible with 3D printing is greatly constrained. Accordingly, the quest for natural ingredients suitable for 3D printing of foods has garnered escalating scholarly interest. In particular, the recent literature has begun to probe the viability of insect proteins as printing substrates. Chitin, a linear polysaccharide composed of β-(1→4)-linked N-acetyl-D-glucosamine units, is a major structural component of the insect exoskeleton. It is chemically distinct from chitosan, which is the partially or fully deacetylated derivative of chitin. In this study, we specifically focus on the native chitin fraction present in the larval powder. As a case in point, Zhang and colleagues elucidated that the protein-to-chitin ratio constitutes the pivotal factor that modulates the 3D printing behavior of edible insect-based gel systems [4]. Among the various types they experimented with, cricket gel showed the best balance between fluidity and self-supporting properties.

Previous studies have mainly focused on improving the adaptability of gel 3D printing by adding an appropriate amount of auxiliary substances [25,26,27]. However, this method dilutes the protein, increases the difficulty of extrusion, and weakens the structural stability, making it unsuitable for 3D printing [28,29,30]. If the tertiary structure of the protein gel can be regulated and the stability of the gel structure can be enhanced without significantly increasing the difficulty of extrusion, this will be more conducive to 3D printing. Some studies have shown that protein gels can improve the printability of composite gels by adding substances such as polyphenols [30]. However, there are few reports on whether excessive addition may have negative effects.

Anthocyanins are a class of plant-derived water-soluble natural pigments and contribute to the rich red, purple, and blue colors of dark-colored fruits such as grapes. Anthocyanins also possess multiple physiological functions, including strong antioxidant and anti-inflammatory activities, as well as protective effects on vision and cardiovascular health. In recent years, with the rapid development of food 3D printing technology, anthocyanins as functional natural pigments have attracted increasing attention [25]. Anthocyanins belong to the flavonoid family and contain multiple phenolic hydroxyl (-OH) groups in their molecular structure as the basis of their biological activity. These structural characteristics make anthocyanins remarkable hydrogen donors capable of forming hydrogen bonds with protein acceptors [26,27]. Through non-covalent interactions with proteins, anthocyanins may play a key role in protein gel modification [28]. It has been reported that blueberry anthocyanins enhance the interaction between ovalbumin and cassava starch [29], and that non-covalent interactions formed through hydrogen bonding between blueberry anthocyanins and potato starch improve gel flowability and support stability [30]. Therefore, grape seed anthocyanin (GSA) was selected in this study as a representative case for interaction with black soldier fly protein (BSFP).

Although these studies demonstrated the modifying potential of anthocyanins on protein gels and the feasibility of using insect proteins in 3D printing, to our knowledge no systematic research on the blackfly protein–anthocyanin composite system in the field of 3D printing has been reported. Key issues that remain for investigation include how anthocyanins affect the 3D printing suitability of blackfly protein gels by regulating their rheological behavior, water state, protein secondary structure, and micro-network structure, as well as the effects and optimal ratios of different amounts of anthocyanin.

Therefore, in this study, BSFP was used as the base material, and composite gels were prepared by adding 0%, 1%, 2%, 3%, 4%, and 5% of GSA. A systematic investigation was conducted with rheometers, FTIR, low-field NMR, and SEM. The focus was placed on how the GSA content modulates the rheology, protein secondary structure, water state, microstructure, and 3D printing performance of the composite gels. The aim was to reveal the intrinsic relationship between the above physicochemical properties and 3D printing performance, to determine the minimum effective GSA concentration, to improve the print quality of BSFP gels, and to find a balance between performance and economic feasibility. This study provides a theoretical basis for enhancing the 3D printability of insect-derived protein gels through flavonoids and opens up new paths for the application of insect protein in the food industry.

## 2. Materials and Methods

### 2.1. Materials

BSFP is a complete dry powder from the larvae of the black soldier fly (*Hermetia illucens*) after drying and grinding. It was purchased from Qingdao Sino Crown Biological Engineering Co., Ltd. (Qingdao, China; http://www.qdsinocrown.com/ (accessed on 3 October 2025)). Its protein content was 24.5% (*w*/*w*), and its chitin content was 6.7% (*w*/*w*). GSA (98%, *w*/*w*) was purchased from Lanzhou Water Rice Biotechnology Co., Ltd. (Lanzhou, China, www.wtlsb.com (accessed on 15 January 2026)). All deionized water used in this work was produced by a Milli-Q^®^ IQ 7000 system (Merck KGaA, Darmstadt, Germany).

### 2.2. 3D Printing Inks

BSFP powder was crushed and passed through an 80-mesh sieve. Based on preliminary experiments, the optimal formulation of the BSFP gel for 3D printing was determined to have a moisture content of 410% (*w*/*w*) on a dry basis. Using this optimal BSFP gel formulation as the control group, GSA was added at concentrations of 1–5% (*w*/*w*) in 1% increments. The mixtures were gently stirred (4500 rpm for 10 min) at room temperature (25 °C) to prepare BSFP-GSA composite gels with different GSA concentrations. Finally, the samples were stored at 4 °C for 12 ± 2 h to allow gel formation.

### 2.3. FTIR Spectroscopy

FT-IR data were recorded on a Nicolet iS20 spectrometer from Thermo Fisher Scientific (Waltham, MA, USA). Lyophilized samples were homogenized with KBr (Sigma-Aldrich, St. Louis, MO, USA; CAS 7758-02-3; IR grade) at a mass ratio of 1:100, and the resulting powders were pressed into pellet form after further grinding. The spectral window was set from 500 to 4000 cm^−1^ with a 4 cm^−1^ step size, and 32 individual scans per sample were accumulated and averaged [31]. All measurements were performed under ambient conditions (25 °C).

### 2.4. LF-NMR

The determination of water status in the gel systems was carried out by LF-NMR on a MicroMR12-025V instrument (Niumag, Shanghai, China), in accordance with the approach proposed by Zheng et al. [32]. The operating parameters were set as follows: relaxation delay = 3500 ms, echo delay = 0.500 ms, echo count = 4000, scan accumulation = 4, points per echo = 200,002, and detection bandwidth = 100 kHz.

### 2.5. Microstructure Morphology

Morphological inspection of the gel specimens was conducted on a ZEISS Sigma 300 SEM (Zeiss, Oberkochen, Germany), following the protocol established by Lopez-Sanchez et al. [33]. Prior to observation, the gel samples were maintained at −80 °C for 12 h and then subjected to freeze drying. The lyophilized materials were subsequently cut into cuboids of 5 mm × 5 mm × 2 mm for microscopic examination.

### 2.6. Rheology

Prior to measurement, the gel samples were removed from refrigerated storage and conditioned at 25 °C. All rheological data were acquired on a DHR-2 rheometer (New Castle, DE, USA) with a 25 mm parallel-plate geometry and maintained at the same temperature. The gap distance was adjusted to 1 mm in order to avoid slippage, and each specimen was allowed to rest for 1 min post-loading to eliminate any residual stress from sample introduction [34]. Strain-sweep tests with strain amplitudes from 0.01% to 10,000% were performed to identify the linear viscoelastic region.

#### 2.6.1. Dynamic Viscosity

Dynamic viscosity was computed in accordance with Wang et al.’s method [35] over a shear-rate window of 0.1–100 s^−1^, and the experimental points were fitted using the Bird–Carreau law (Model (1)).

(1) $$\eta =\eta_{\infty }+(\eta_{0}-\eta_{\infty }){\left[1+{\left(\lambda \dot{\gamma }\right)}^{2}\right]}^{\frac{n-1}{2}}$$

where η is the viscosity (Pa⋅s), η_∞_ is the viscosity at an infinite shear rate (Pa⋅s), η_0_ is the viscosity at a zero shear rate (Pa⋅s), λ is the relaxation time (s), γ˙ is the shear rate (s^−1^) and n is the flow behavior index.

#### 2.6.2. Frequency Sweep

Frequency sweep experiments were employed at a strain level of 1% within the linear viscoelastic zone, spanning an angular frequency range of 0.1 to 100 rad/s, as described in Guo et al. [36].

#### 2.6.3. Stress Amplitude Sweep

Oscillatory stress amplitude sweeps were conducted at a frequency of 1 Hz, spanning stress levels from 1 to 4000 Pa.

#### 2.6.4. Creep

Each sample was subjected to a creep test under a constant stress of 50 Pa for 300 s. Sample deformation was monitored throughout the test.

### 2.7. pH Measurement

The pH of the composite gel samples in all groups was determined at 25 °C with a pH meter (model PB-30, Sartorius, Göttingen, Germany).

### 2.8. 3D Printing

A FOODBOT-D1 3D printer (Hangzhou Shiyin Technology Co., Ltd., Hangzhou, China) was used to assess the print fidelity and mechanical stability of the gel samples. The printing configuration comprised a speed of 30 mm/s, a piston advancement rate of 0.04 mm/s, and a 1.2 mm nozzle diameter. Fidelity was quantified according to the methodology of Guo et al. [37] by printing a 20 mm × 20 mm × 4 mm model containing four holes of 6 mm × 6 mm × 4 mm. Structural stability was characterized through the printing of a hollow cylinder of 17.35 mm in diameter and 70.00 mm in height. Visual documentation of the printed objects was performed with an iPhone 12 (Apple, Cupertino, CA, USA) equipped with a 120-megapixel imaging system (aperture f/1.6). ImageJ 1.52a (NIH, Bethesda, MD, USA) was used for image analysis of the printed objects. The parameters used to evaluate the printing fidelity included the hole’s area, the length of the longest side, and print fidelity. Print fidelity was calculated as follows:

(2) $$Printing\;fidelity=\left(1-\frac{\left|printed\;area-model\;area\right|}{model\;area}\right)\times 100\%$$

### 2.9. Statistical Analysis

Unless otherwise specified, all experiments used three independent biological replicate samples, and the data from each replicate sample represented the average of at least three technical replicate samples. Statistical analyses were performed using one-way ANOVA, and all values are given as means ± standard deviations. Intergroup differences were assessed at a significance level of 0.05 using SPSS (version 23, SPSS Inc., Chicago, IL, USA). Data fitting for apparent viscosity and creep response was carried out with OriginPro 2021 (OriginLab, Northampton, PA, USA), employing the Bird–Carreau law model for viscosity.

## 3. Results and Discussion

### 3.1. Effect of GSA on the Secondary Structure of Composite Gels

**Figure 1:**
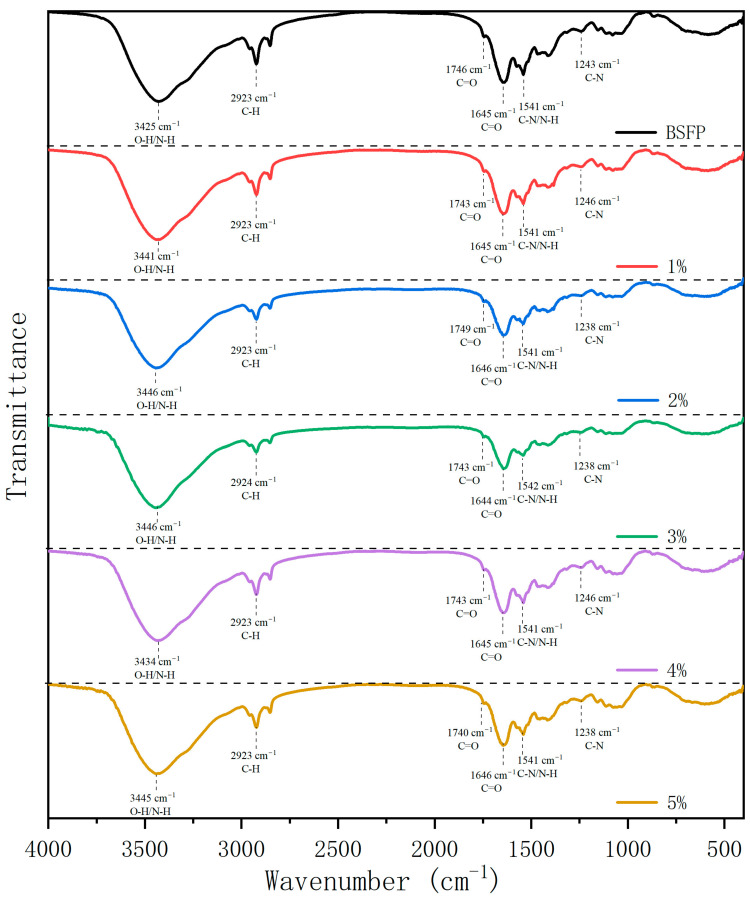
FT-IR spectra of BSFP-GSA gels with different formulations.

FTIR analysis serves as a powerful tool for elucidating the functional groups and chemical bonds in food matrices. The spectra of composite gels as a function of GSA concentration are depicted in Figure 1. Overall, all samples exhibited the same absorption bands, but with differences in intensity, indicating that the addition of GSA did not generate new spectral bands and that physical interactions occurred among the polymers in the gels.

The amide I band falls within the wavenumber range of 1644.98–1648.84 cm^−1^ and originates predominantly from C=O stretching vibrations [38]. With increasing GSA content, the absorption peak of the composite gels near 1640 cm^−1^ showed a rightward shift, indicating that anthocyanin addition disrupted some of the internal hydrogen bonds within the protein.

The amide A band falls within the 3600–3200 cm^−1^ range and originates from O–H stretching and N–H stretching, as well as hydrogen-bond stretching [39]. As the GSA level increased, the absorption peak of the composite gels near 3300 cm^−1^ first shifted left and then right, moving from 3324.96 cm^−1^ to 3418.69 cm^−1^ and then to 3441.35 cm^−1^, while the absorbance value increased. This suggests that the addition of an appropriate concentration of GSA strengthened inter- and intra-molecular hydrogen bonding and promoted stronger hydrogen bonds between proteins and water. This may be due to enhanced stability in the functional groups and increased intermolecular interactions [40]. However, excessive GSA addition to proteins can induce molecular polarization and disrupt the non-covalent interactions that maintain the protein’s spatial structure, thereby leading to unfavorable protein aggregation.

**Figure 2:**
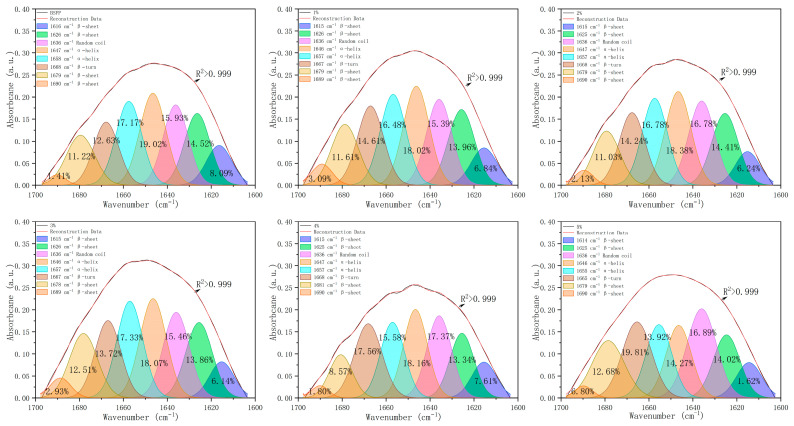
Secondary protein structure of BSFP-GSA gels with different formulations.

The secondary structures of BSFP and the composite gels were analyzed by deconvoluting the amide I band located at 1600–1700 cm^−1^ (Figure 2). The addition of GSA altered the composition of secondary structural elements in the composite gels: with increasing anthocyanin addition, the beta-turn content increased and the alpha-helix content decreased. The effects of different GSA ratios on beta sheets and random coil structures varied. Changes to the type and proportion of the protein’s secondary structure reflect variations in its structural stability. In the composite gels with different GSA concentrations, the contents of beta turns and random coils were lower at low GSA concentrations than at high concentrations. Among all samples, the composite gel containing 3% GSA exhibited the lowest random coil content (15.46%) and beta-turn content (13.72%).

The addition of 3% GSA reduced the content of disordered structures (random coils and beta turns), which was beneficial for stabilizing the composite gel structure. In contrast, the addition of high-level GSA elicited molecular polarization, which perturbed the ensemble of non-covalent interactions (hydrophobic, van der Waals, electrostatic, and hydrogen bonding) that sustain the protein tertiary structure. The perturbation culminated in conformational transitions and a marked rise in structural randomness. Similar phenomena have been observed when anthocyanins were added to soy protein isolates, rice protein, and whey protein systems [41,42,43].

### 3.2. Effect of GSA on the Water State of BSFP Gels

**Figure 3:**
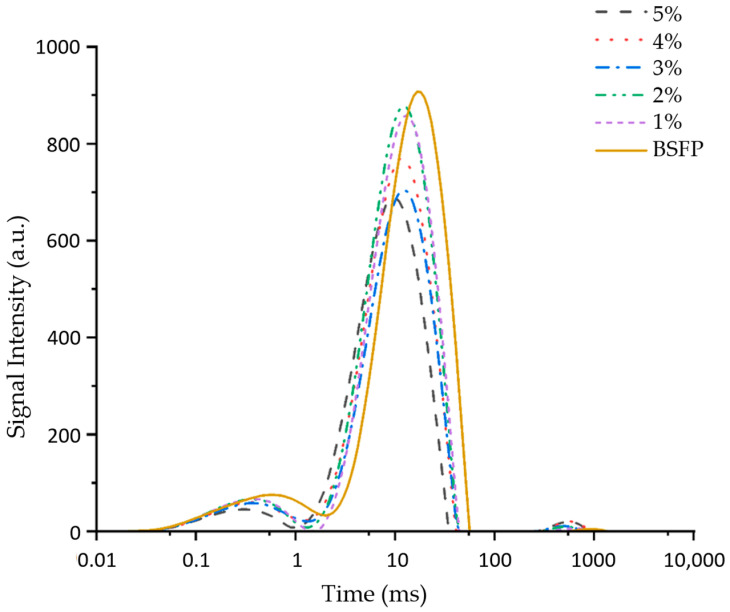
Relaxation time curves of BSFP-GSA gels with different formulations.

The state of water within the composite gels was evaluated by LF-NMR, with the transverse relaxation times (T_2_) of the different gel formulations visualized in Figure 3. The water distribution data can be associated with the rheological characteristics of the gels, thereby accounting for their performance in 3D printing applications. It is commonly accepted that restricted water mobility is attributable to the generation of a stronger gel network of the sample, which in turn affects its rheological properties and microstructure [44].

As shown in Figure 3, T22 (immobilized water) accounted for the biggest proportion among all water states in the composite gels, implying that it played a dominant role in the rheological properties of the gels [45]. After the addition of GSA, the peak area proportion of T22 in the composite gels increased significantly, indicating that the incorporated anthocyanin promoted changes in water state. This may be attributed to hydrogen bond formation between GSA and the macromolecular polymers in the gel network, resulting in a denser gel network that restricted water mobility [46]. The leftward shift in the T22 peak indicates a shortening of the relaxation time of immobilized water, suggesting that this fraction of water was subjected to stronger physical constraints. The addition of GSA disrupted some of the internal hydrogen bonds in the BSFP, as reflected by the increase in beta-sheet content (Figure 2). This exposed polar groups, such as carbonyl and amino groups, that were originally buried within the hydrophobic core of the protein. These exposed polar groups formed new and stronger hydrogen bonds with surrounding water molecules. Meanwhile, although anthocyanins disrupted the original secondary-structure hydrogen bonds in the proteins, hydrophobic interactions occurred between the aromatic rings of anthocyanins and the hydrophobic groups in the proteins, and anthocyanins formed “bridges” between different protein chains. Figure 3 shows a single dominant T22 peak or a double peak (T21 + T22) following the addition of anthocyanin. The narrower width of the peak indicates that the water molecules were more uniformly distributed within the gels and that the microstructure became more homogeneous.

### 3.3. Effect of GSA on the Microstructure of BSFP Gels

**Figure 4:**
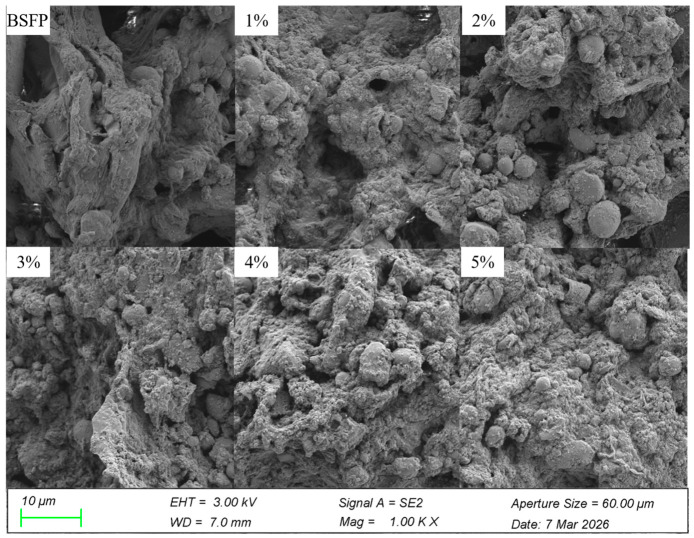
Microstructure of BSFP-GSA gels with different formulations.

The microscopic network structure of BSFP gels critically influences their 3D printing performance. To elucidate this, we examined the microstructure of freeze-dried composite gels with varying GSA levels by scanning electron microscopy. The resulting micrographs are presented in Figure 4.

For the GSA-free control group, the 1000× scanning electron microscopy images showed a continuous, open, three-dimensional network structure, with irregular polygonal pores, a relatively uniform pore size distribution, and fine granular textures on the pore walls under low voltage. Without GSA, such a structure has low mechanical strength with poor self-supporting ability and the risk of collapse during subsequent 3D printing [47]. However, after introducing GSA, the microstructure of the composite gels varied significantly. The network became denser and more compact, with thicker pore walls. The pore size decreased overall and became more uniformly distributed, and the pore wall surfaces changed from a relatively smooth texture to a densely granular texture accompanied by fine wrinkles. These observations indicate that strong interactions occurred between anthocyanins and proteins, inducing protein aggregation and enhancing the compactness of the gel network. The formation of denser network structures has likewise been observed when polyphenols are added to proteins [48,49,50,51,52]. Compared to the control, the newly formed network in the composite gels may act as a skeletal structure, thereby improving the deformation resistance of the gels.

### 3.4. Effect of GSA on the Rheology of BSFP-GSA Composite Gels

#### 3.4.1. Apparent Viscosity

**Figure 5:**
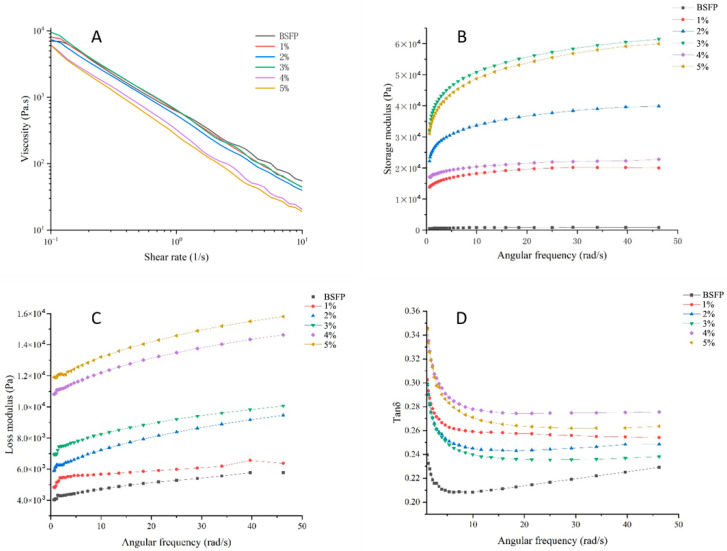
Rheological properties of BSFP-GSA gels with different formulations: (**A**) viscosity, (**B**) storage modulus, (**C**) loss modulus, and (**D**) Tanδ.

Food 3D printing materials should possess sufficiently high viscosity to maintain the shape stability of the printed model while also being sufficiently low in viscosity to allow smooth extrusion through small-diameter nozzles [32]. Therefore, for protein gel samples serving as 3D printing inks, an appropriate apparent viscosity provides the material with suitable flowability and spreadability [53]. The shear-rate dependence of apparent viscosity for the composite gels is depicted in Figure 5A. With η_∞_ fixed at zero, the kinetic parameters extracted from Bird–Carreau law model regression of these viscosity data are presented in Table 1. A decrease in apparent viscosity was observed for all samples as the shear rate increased, indicating that the gel network was disrupted under high shear and exhibited typical shear-thinning behavior. At identical shear rates, the apparent viscosity of composite gels amended with 1–3% GSA surpassed that of the unamended control, whereas gels with 4–5% GSA displayed decreased viscosity. It can be deduced from these results that the introduction of GSA at low dosages (1–3%) enhances the apparent viscosity of the composite gels, thereby endowing them with adequate fluidity and spreading properties. This is expected to enable the gels to flow efficiently through fine-deposition nozzles for extrusion-based 3D printing [54]. Similar results have also been observed in protein gels supplemented with polyphenols [29,30,55]. This may be ascribed to the role of polyphenols in promoting cross-linking within the protein–polyphenol gel ink network structure.

The fitted coefficients of determination (R^2^) ranged from 0.9698 to 0.9905, indicating that the Bird–Carreau model provided an acceptable description of the shear-dependent viscosity behavior of all formulations. The fitted flow behavior index (n) was positive and lower than one, confirming physically meaningful shear-thinning behavior for all samples. Since the flow behavior index (n) was very close to one, this mainly indicates strong shear-thinning behavior within the tested shear-rate range and was not used further for comparisons among formulations.

The zero-shear viscosity (η_0_) increased from 4.57 × 10^5^ Pa·s in the control sample to 6.15 × 10^5^, 7.12 × 10^5^, and 8.81 × 10^5^ Pa·s in the 1%, 2%, and 3% GSA groups, respectively. The highest η_0_ was observed in the 3% GSA group, suggesting that moderate GSA addition enhanced the low-shear viscosity and strengthened the gel network. This may be attributed to enhanced non-covalent interactions between GSA and BSFP, which promoted protein aggregation and network densification. However, further increasing the GSA concentration to 4% and 5% reduced η_0_ to 7.11 × 10^5^ and 6.84 × 10^5^ Pa·s, indicating that excessive GSA did not further improve the viscosity of the composite gels. This may be related to excess anthocyanin adsorbing onto the protein network surface, thereby weakening effective interchain friction and reducing the viscosity contribution of the gel network.

The relaxation time (λ) showed a similar trend, increasing from 1093.95 s in the control to 1428.71 s in the 3% GSA group, followed by a decrease at higher GSA concentrations. This shows that moderate GSA addition prolonged the structural relaxation of the gel network, whereas excessive GSA weakened the network response under shear deformation. Consistent outcomes were reported by Sun et al. [34] and Luo et al. [56] in investigations of polysaccharide-enriched soybean protein matrices. Overall, the 3% GSA formulation exhibited the most favorable rheological behavior, with the highest low-shear viscosity and strong shear-thinning characteristics. This rheological profile is beneficial for extrusion-based 3D printing, as it allows the material to maintain shape stability after deposition while becoming easier to extrude under high-shear conditions during nozzle flow.

#### 3.4.2. Storage Modulus and Loss Modulus

As a preliminary step to the frequency sweeps, oscillatory amplitude sweeps were executed to delineate the linear viscoelastic regime for every formulation. As summarized in Table 1, the critical stress (σ LVR)—defined as the stress at which G′ decreases by 5% from its plateau value—was determined for each sample. It is worth noting that the amplitude sweep results confirmed that the applied creep stress of 50 Pa was less than critical stress corresponding to a 5% deviation for all samples, ensuring that the subsequent creep tests were conducted strictly within the LVR. Under these validated linear conditions, the effects of GSA addition on the storage (G′) and loss (G″) moduli of the composite gels were investigated, and the results are shown in Figure 5B,C. At a given angular frequency, the addition of GSA increased both G′ and G″, indicating that anthocyanin incorporation promoted the strength and structural stability of the composite gels. Similarly, previous studies found that the addition of EGCG increased the G′ and G″ values of quinoa protein isolate systems and promoted the assembly of elastic gel-like emulsion networks [57].

To characterize the rheological behavior of BSFP-GSA composite gels, correlation analysis was performed using tanδ—defined as the ratio of G″ to G′ and indicating the gel’s plasticity. As shown in Figure 5D, the tanδ values of all composite gels were greater than that of the control BSFP gel, demonstrating that GSA increased the plasticity of the BSFP gel, which is beneficial for shape formation during 3D printing [36]. Cross-linking between proteins and anthocyanins at high anthocyanin concentrations, together with the filling effect of anthocyanin self-aggregates within the gel network, increased both the strength and the number of network cross-linking points. Therefore, although high anthocyanin addition reduced the apparent viscosity of the gels, both the storage modulus and loss modulus remained higher than those of the control.

#### 3.4.3. Creep Properties

**Figure 6:**
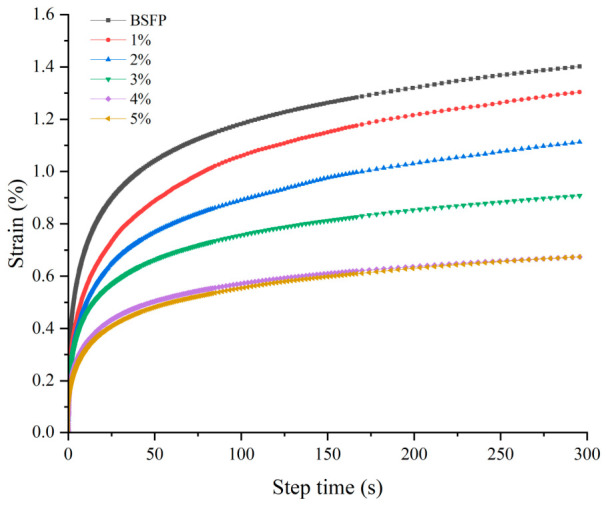
Creep curves of BSFP-GSA gels with different formulations.

Creep refers to time-dependent deformation under constant stress [58] and is used to assess the deformation resistance of printing materials. Compared to the BSFP gel, the composite gels exhibited a certain degree of strain, and this strain decreased with increasing GSA content (Figure 6). This indicates that the addition of GSA enhanced the deformation resistance and rigidity of the gels. Similarly, it has been reported that polyphenolic substances can enhance the rigidity of protein gels, possibly due to the formation of dense aggregates that improve the cohesion and elasticity of the gel network [59,60].

#### 3.4.4. Effect of GSA on the pH of Composite Gels

With increasing GSA addition, the pH of the composite gels decreased gradually from 8.55 ± 0.06 to 8.15 ± 0.11, which is attributable to proton release from the phenolic hydroxyl groups of GSA (Table 2). Despite this slight decline, all formulations remained within a narrow alkaline window (ΔpH ≈ 0.4 units), which is insufficient to induce extensive protein denaturation or charge reversal near the isoelectric point (pI ≈ 4.5–5.0). Therefore, the pronounced improvements in G′, yield stress, and shear-thinning behavior—and consequently the superior 3D printing performance—cannot be primarily ascribed to pH effects. Instead, they arise from direct non-covalent cross-linking (hydrogen bonds and hydrophobic interactions) between GSA and BSFP. The mild pH reduction may play a permissive auxiliary role by subtly favoring hydrogen bond formation, but the dominant driving force is GSA-BSFP association, as further supported by FTIR and rheological evidence.

### 3.5. Effect of GSA on the 3D Printing Suitability of the Composite Gels

**Figure 7:**
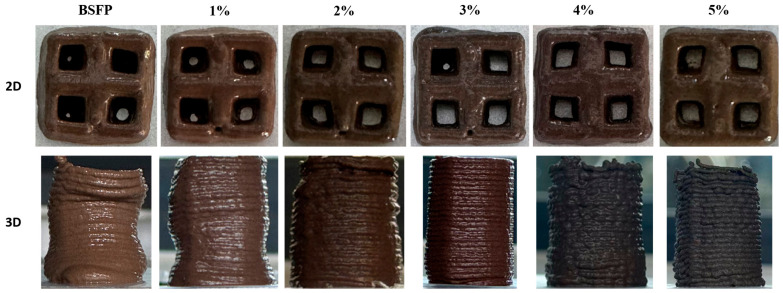
3D printing performance of BSFP-GSA gels with different formulations.

The printing performance of the control and composite gel inks was evaluated by 3D printing. In general, a well-printed product should have a smooth surface, uniform lines, and a stable structure. The 3D printing outcomes of the control and composite gels are shown in Figure 7, and the detailed data are listed in Table 3.

No significant differences were observed in the hole’s area or in the length of the longest side among all gels. However, significant differences were found in printing fidelity: all anthocyanin-containing gels showed significantly higher printing fidelity than the control, and gels containing 3–5% GSA showed significantly higher printing fidelity than those containing 1–2% GSA. Assessment of the structural robustness of the printed constructs indicated that hollow cylinders derived from the control and 1–2% GSA groups underwent partial sagging, while those from the 3–5% GSA groups manifested pronounced self-sustaining properties. This observation points to the superior structural stability of the composite gels over the control. As a cross-linking agent, GSA can form a more complex and stable network with proteins, thereby improving the structure and properties of BSFP [36]. To contextualize our fidelity data, the 71.05–75.66% achieved here is higher than the range obtained from unmodified insect gels [4] and comparable to polyphenol-modified plant protein gels [30,36], confirming that GSA effectively enhances BSFP printability to a level similar to that of optimized plant-based inks [37]. According to the principle of achieving optimal performance with minimum effective addition, 3% GSA addition was confirmed as the optimal formulation for the BSFP-GSA composite gel.

The BSFP used in this study was whole-insect powder, which retained the natural components of the raw insect materials, including chitin. Since all the formulations used the same batch of whole-insect powder, the concentrations of chitin and other non-protein components were constant among the groups. Thus the relative comparison of the GSA addition effect was not affected. Previous studies have shown that the ratio of protein to chitin is a key factor influencing the 3D printing behavior of insect gels [4]. Chitin, as an insoluble dietary fiber, may provide physical filling in the gel network or form a composite network with proteins to enhance structural stability, which may have potential contributions to the self-supportability of 3D printing [4]. However, the systematic rheological and microstructural changes observed in this study along with variations in the concentration of GSA (such as increased G’ and G”, reduced pore size, densification of the network, etc.) were mainly driven by non-covalent interactions between anthocyanins and proteins. Although the potential hydrogen bond interactions between chitin and anthocyanins could not be completely ruled out, they were secondary factors in this system. This strategy of using all components is also more in line with the sustainable concept of high-value utilization of insect resources: without the need for additional purification steps, it reduces processing costs and energy consumption.

## 4. Conclusions

This study systematically investigated the rheological properties, water content, secondary protein structure, microstructure, and 3D printing adaptability of the BSFP-GSA composite gel. The results showed that 1–3% of GSA could improve the printing performance, with 3% being the optimal ratio. Specifically, the printing fidelity increased from 45.73% (control group) to 71.05% (3% GSA). The 3% GSA group also exhibited the highest content of ordered secondary structures (β-sheets increased to 35.43%; disordered coiled structures decreased to 15.46%). LF-NMR and SEM analyses confirmed that the gel network became more dense, the water molecule fixation ability was enhanced, and the pore size was reduced. However, excessive addition (4–5%) causes the anthocyanin to adsorb onto the protein network skeleton surface, reducing the friction between chain segments. This results in a decrease in apparent viscosity that is detrimental to printing. As an insect protein-based 3D printing food ink, the composite gel has both nutritional and printable properties. It is suitable for personalized nutrition customization, functional foods, and the development of sustainable protein substitute products. Anthocyanin provides natural color and antioxidant properties, improves processing adaptability, and helps alleviate sensory resistance to insect food by consumers. Future research should focus on batch stability, structural evolution during storage, and sensory acceptance and further evaluate its practical application potential in combination with food sensory and nutrition science.

## Figures and Tables

**Table 1:** Fitted Bird–Carreau law model coefficients of BSFP-GSA gels with different formulations.

	η_0_ (Pa·s)	λ (s)	n	R^2^
BSFP	4.57 × 10^5^	1093.95	2.34 × 10^−14^	0.9864
1%	5.16 × 10^5^	1220.86	2.22 × 10^−14^	0.9905
2%	7.12 × 10^5^	1298.06	2.22 × 10^−14^	0.9891
3%	8.81 × 10^5^	1428.71	2.22 × 10^−14^	0.9821
4%	7.11 × 10^5^	1348.13	2.22 × 10^−14^	0.9732
5%	6.84 × 10^5^	1156.91	2.28 × 10^−14^	0.9698

**Table 2:** pH of BSFP-GSA gels with different formulations.

GSA	0%	1%	2%	3%	4%	5%
pH	8.55 ± 0.06	8.46 ± 0.01	8.42 ± 0.04	8.40 ± 0.03	8.30 ± 0.04	8.15 ± 0.11

**Table 3:** Printing fidelity of BSFP-GSA gels with different formulations.

	Hole Area (mm^2^)	Longest Side Length (mm)	Printing Fidelity (%)
BSFP	121.23 ± 5.63 ^a^	23.73 ± 2.19 ^a^	45.73 ± 3.15 ^c^
1%	123.87 ± 3.90 ^a^	23.03 ± 3.52 ^a^	60.08 ± 3.06 ^b^
2%	127.46 ± 4.48 ^a^	22.99 ± 2.92 ^a^	62.14 ± 3.14 ^b^
3%	126.46 ± 3.90 ^a^	22.17 ± 2.53 ^a^	71.05 ± 2.61 ^a^
4%	118.33 ± 4.48 ^a^	22.22 ± 2.54 ^a^	72.78 ± 2.20 ^a^
5%	123.19 ± 3.88 ^a^	21.39 ± 1.57 ^a^	75.66 ± 1.47 ^a^

Note: Different letters in the same column indicate that mean values are significantly different (*p* < 0.05).

## Data Availability

The original contributions presented in this study are included in the article. Further inquiries can be directed to the corresponding author.

## References

[1] Ochiai M., Tezuka K., Yoshida H., Akazawa T., Komiya Y., Ogasawara H., Adachi Y., Nakada M. (2022). Edible Insect Locusta migratoria Shows Intestinal Protein Digestibility and Improves Plasma and Hepatic Lipid Metabolism in Male Rats. Food Chem..

[2] Vauterin A., Steiner B., Sillman J., Kahiluoto H. (2021). The Potential of Insect Protein to Reduce Food-Based Carbon Footprints in Europe: The Case of Broiler Meat Production. J. Clean. Prod..

[3] Pilco-Romero G., Chisaguano-Tonato A.M., Herrera-Fontana M.E., Chimbo-Gándara L.F., Sharifi-Rad M., Giampieri F., Battino M., Vernaza M.G., Álvarez-Suárez J.M. (2023). House Cricket (*Acheta domesticus*): A Review Based on Its Nutritional Composition, Quality, and Potential Uses in the Food Industry. Trends Food Sci. Technol..

[4] Zhang W., Jia Y., Guo C., Devahastin S., Hu X., Yi J. (2024). Effect of Compositions and Physical Properties on 3D Printability of Gels from Selected Commercial Edible Insects: Role of Protein and Chitin. Food Chem..

[5] Malla N., Nørgaard J.V., Lærke H.N., Heckmann L.-H.L., Roos N. (2022). Some Insect Species Are Good-Quality Protein Sources for Children and Adults: Digestible Indispensable Amino Acid Score (DIAAS) Determined in Growing Pigs. J. Nutr..

[6] Tomberlin J.K., Adler P.H., Myers H.M. (2009). Development of the Black Soldier Fly (Diptera: Stratiomyidae) in Relation to Temperature. Environ. Entomol..

[7] Malla N., Opeyemi A.J. (2018). Prospects of Insects as Alternative Protein Source: Broiler Chicken and Growing Pigs. Sustain. Anim. Nutr. Feed..

[8] Liu X., Chen X., Wang H., Yang Q., Rehman K.U., Li W., Cai M., Li Q., Mazza L., Zhang J. (2017). Dynamic Changes of Nutrient Composition throughout the Entire Life Cycle of Black Soldier Fly. PLoS ONE.

[9] Reuben S.J., Chandra S., Praveena B., Santhoshkumar P., Moses J.A. (2025). 3D printing of alternative proteins: Approaches, challenges and advances. Sustain. Mater. Technol..

[10] Giacalone D., Jaeger S.R. (2023). Consumer acceptance of novel sustainable food technologies: A multi-country survey. J. Clean. Prod..

[11] Herdeiro F.M., Carvalho M.O., Nunes M.C., Raymundo A. (2024). Development of Healthy Snacks Incorporating Meal from *Tenebrio molitor* and *Alphitobius diaperinus* Using 3D Printing Technology. Foods.

[12] Su S., Pilavci E., Yesiloz Ş.E., Demir A.U., Sancakli A., Gunduz S., Kibar E.A.A., Bahar M.B., Gunduz O. (2025). An innovative approach of 3D printed functional foods: Grasshoppers based cookies. Int. J. Gastron. Food Sci..

[13] Dankar I., Haddarah A., Omar F.E.L., Sepulcre F., Pujolà M. (2018). 3D Printing Technology: The New Era for Food Customization and Elaboration. Trends Food Sci. Technol..

[14] Liu Z., Zhang M., Bhandari B., Wang Y. (2017). 3D Printing: Printing Precision and Application in Food Sector. Trends Food Sci. Technol..

[15] Sun J., Zhou W., Huang D., Fuh J.Y.H., Hong G.S. (2015). An Overview of 3D Printing Technologies for Food Fabrication. Food Bioprocess Technol..

[16] Zhong Y., Zeng S., Lv Y., Lv W., Xiao H., Sheng S. (2024). Effect of Guar Gum on the Rheological Properties, Microstructure and 3D Printing Performance of Egg Yolk Powder-Potato Starch Composite Gel. Food Hydrocoll..

[17] Kadival A., Kour M., Meena D., Mitra J. (2023). Extrusion-Based 3D Food Printing: Printability Assessment and Improvement Techniques. Food Bioprocess Technol..

[18] Xu B., Wang X., Chitrakar B., Xu Y., Wei B., Wang B., Lin L., Guo Z., Zhou C., Ma H. (2025). Effect of Various Physical Modifications of Pea Protein Isolate (PPI) on 3D Printing Behavior and Dysphagia Properties of Strawberry-PPI Gels. Food Hydrocoll..

[19] Li G., Wang B., Yang L., Lv W., Xiao H. (2025). Effect of Salt Valence and Ionic Strength on the Rheology and 3D Printing Performance of Walnut Protein Emulsion Gels. Food Hydrocoll..

[20] Chen Y., Zhang M., Sun Y., Phuhongsung P. (2022). Improving 3D/4D Printing Characteristics of Natural Food Gels by Novel Additives: A Review. Food Hydrocoll..

[21] He C., Zhang M., Fang Z. (2020). 3D Printing of Food: Pretreatment and Posttreatment of Materials. Crit. Rev. Food Sci. Nutr..

[22] Hussain S., Malakar S., Arora V.K. (2022). Extrusion-Based 3D Food Printing: Technological Approaches, Material Characteristics, Printing Stability, and Post-Processing. Food Eng. Rev..

[23] Jiang H., Zheng L., Zou Y., Tong Z., Han S., Wang S. (2019). 3D Food Printing: Main Components Selection by Considering Rheological Properties. Crit. Rev. Food Sci. Nutr..

[24] Feng C., Zhang M., Bhandari B. (2019). Materials Properties of Printable Edible Inks and Printing Parameters Optimization during 3D Printing: A Review. Crit. Rev. Food Sci. Nutr..

[25] Tan G., Hou J., Meng D., Zhang H., Han X., Li H., Wang Z., Ghamry M., Rayan A.M. (2024). 3D Printing Cassava Starch-Ovalbumin Intelligent Labels: Co-Pigmentation Effects of Gallic Acid on Anthocyanins. Int. J. Biol. Macromol..

[26] Gao R., Song R., Shen L., Zhao X., Xue L., Li J., Zheng X. (2023). Evaluation of 3D Printability of Blueberry Powder Gel System under Ultrasound Pretreatment. LWT.

[27] Li Y., Li J., Xiong R., Lu R., Hossen M.A., Dai J., Li S., Qin W., Liu Y. (2021). Three-Dimensional Printed Mulberry Anthocyanin Combined with Chitosan/Hydroxyethyl Cellulose Bilayer Films for Quality Preservation of Litchi. SSRN.

[28] Li S., Jiang Y., Zhou Y., Li R., Jiang Y., Hossen M.A., Dai J., Qin W., Liu Y. (2022). Facile Fabrication of Sandwich-like Anthocyanin/Chitosan/Lemongrass Essential Oil Films via 3D Printing for Intelligent Evaluation of Pork Freshness. Food Chem..

[29] Bao Y., Wang M., Si X., Li D., Gui H., Jiang Q., Li J., Yang S., Yang Y., Li Z. (2024). Customized Development of 3D Printed Anthocyanin-Phycocyanin Polychromatic Oral Film via Chondroitin Sulfate Homeostasis: A Platform Based on Starch and κ-Carrageenan. Carbohydr. Polym..

[30] Zhang W., Yi J., Hu X., Du M., Guo C. (2025). A strategy to improve 3D printing performance: Interaction between epigallocatechin gallate (EGCG) and honey bee pupa protein (HBPP). Food Hydrocoll..

[31] Yu H.-Z., Chi S.-Y., Li D., Wang L.-J., Wang Y. (2022). Effect of gums on the multiscale characteristics and 3D printing performance of potato starch gel. Innov. Food Sci. Emerg. Technol..

[32] Zheng Z., Zhang M., Liu Z. (2021). Investigation on evaluating the printable height and dimensional stability of food extrusion-based 3D printed foods. J. Food Eng..

[33] Lopez-Sanchez P., Wang D., Zhang Z., Flanagan B., Gidley M.J. (2016). Microstructure and Mechanical Properties of Arabinoxylan and (1,3;1,4)-β-Glucan Gels Produced by Cryo-Gelation. Carbohydr. Polym..

[34] Sun F., Xu J., Wang Z., Cheng T., Wang D., Liu J., Guo Z., Wang Z. (2024). Effect of glycosylation on soy protein isolate–sodium carboxymethyl cellulose conjugates heat-induced gels and their applications as carriers of riboflavin. Food Hydrocoll..

[35] Wang J., Jiang Q., Huang Z., Muhammad A.H., Gharsallaoui A., Cai M., Yang K., Sun P. (2024). Rheological and mechanical behavior of soy protein-polysaccharide composite paste for extrusion-based 3D food printing: Effects of type and concentration of polysaccharides. Food Hydrocoll..

[36] Guo C., Zhang M., Devahastin S. (2021). Improvement of 3D Printability of Buckwheat Starch-Pectin System via Synergistic Ca^2+^-Microwave Pretreatment. Food Hydrocoll..

[37] Guo C., Zhang M., Devahastin S. (2021). Color/Aroma Changes of 3D-Printed Buckwheat Dough with Yellow Flesh Peach as Triggered by Microwave Heating of Gelatin-Gum Arabic Complex Coacervates. Food Hydrocoll..

[38] Chen Q., Ji H., Wang Z., Wang Y., Wang X., Chen Z. (2025). Effects of different charged polysaccharides on the gelation properties and in vitro digestibility of potato protein gel: Insight into underlying mechanisms. Food Hydrocoll..

[39] Cai L., Feng J., Regenstein J., Lv Y., Li J. (2017). Confectionery gels: Effects of low calorie sweeteners on the rheological properties and microstructure of fish gelatin. Food Hydrocoll..

[40] Han Z.T., Long W.M., Zhang T.H., Dong Z.Y., Yan J.X. (2022). Application of xanthan gum and konjac gum to improve the texture, rheological properties and microstructure of Oviductus Ranae gel. Int. J. Biol. Macromol..

[41] Tang S., Si X., Zang Z., Gui H., Xie X., Wang L., He Y., Yang B., Li B. (2024). Mildly preheating induced conformational changes of soy protein isolates contributed to the binding interaction with blueberry anthocyanins for stabilization. Food Hydrocoll..

[42] Yang Y., Chen L., Chen M., Liu F., Zhong F. (2025). Interactions between rice protein and anthocyanin with different pH-cycle: Structural characterization, binding mechanism and stability. Food Hydrocoll..

[43] Wang Y., Wang S., Zhang X., Wu W., Bai W., Tian L. (2024). Non-covalent interactions of roselle anthocyanins with milk proteins and egg white protein. Food Hydrocoll..

[44] Yang F., Zhang M., Prakash S., Liu Y. (2018). Physical properties of 3D printed baking dough as affected by different compositions. Innov. Food Sci. Emerg. Technol..

[45] Dekkers B.L., de Kort D.W., Grabowska K.J., Tian B., Van As H., van der Goot A.J. (2016). A combined rheology and time domain NMR approach for determining water distributions in protein blends. Food Hydrocoll..

[46] Alvarez M.D., Canet W., Cuesta F., Lamua M. (1998). Viscoelastic characterization of solid foods from creep compliance data: Application to potato tissues. Z. Leb. Und-Forsch. A.

[47] Zhong Y., Wang B., Lv W., Li G., Lv Y., Cheng Y. (2024). Egg yolk powder-starch gel as novel ink for food 3D printing: Rheological properties, microstructure and application. Innov. Food Sci. Emerg. Technol..

[48] Chen J., Chai J., Chen X., Huang M., Zeng X., Xu X. (2023). Development of edible films by incorporating nanocrystalline cellulose and anthocyanins into modified myofibrillar proteins. Food Chem..

[49] Raychaudhuri R., Pandey A., Das S., Nannuri S.H., Joseph A., George S.D., Vincent A.P., Mutalik S. (2021). Nanoparticle impregnated self-supporting protein gel for enhanced reduction in oxidative stress: A molecular dynamics insight for lactoferrin-polyphenol interaction. Int. J. Biol. Macromol..

[50] Guo Z., Huang J., Mei X., Sui Y., Li S., Zhu Z. (2024). Noncovalent conjugates of anthocyanins to wheat gluten: Unraveling their microstructure and physicochemical properties. Foods.

[51] Ren C., Quan T., Li B. (2023). Understanding the effect of anthocyanin-rich extract on the gel and digestive properties of soy protein cold-set gels. Food Biophys..

[52] Seddiek A.S., Chen K., Zhou F., Esther M.M., Elbarbary A., Golshany H., Uriho A., Liang L. (2025). Whey protein hydrogels and emulsion gels with anthocyanins and/or goji oil: Formation, characterization and in vitro digestion behavior. Antioxidants.

[53] Zhang L., Zaky A.A., Zhou C., Chen Y., Su W., Wang H., Abd El-Aty A.M., Tan M. (2022). High internal phase Pickering emulsion stabilized by sea bass protein microgel particles: Food 3D printing application. Food Hydrocoll..

[54] Ji Y., Han C., Liu E., Li X., Meng X., Liu B. (2022). Pickering emulsions stabilized by pea protein isolate-chitosan nanoparticles: Fabrication, characterization and delivery EPA for digestion in vitro and in vivo. Food Chem..

[55] Bai Y., Liu Y., Yao T., Huang X., Wang Y., Qi H. (2024). Synergistic modification of phycocyanin composite gel by xanthan gum and flaxseed gum and the fate during in vitro digestion. Food Hydrocoll..

[56] Lou F., Guo Y., Zhang S., Sun F., Liu J., Jiang L., Guo Z., Wang Z. (2025). Synergistic enhancement of 3D printing performance in soybean protein isolate gel inks by lonic/nonionic polysaccharides: A network rearrangement mechanism revealed by rheology and the sequence of physical processes analysis. Food Hydrocoll..

[57] He X., Yang W., Zhao Q., Qin X. (2023). Controlled oxidation and digestion of Pickering emulsions stabilized by quinoa protein and (-)-epigallocatechin-3-gallate (EGCG) hybrid particles. Int. J. Biol. Macromol..

[58] Guo J., Zhang M., Adhikari B., Ma Y., Luo Z. (2023). Formulation and characterization of 3D printed chickpea protein isolate-mixed cereal dysphagia diet. Int. J. Biol. Macromol..

[59] Chen D., Zhu X., Ilavsky J., Whitmer T., Hatzakis E., Jones O.G., Campanella O.H. (2021). Polyphenols Weaken Pea Protein Gel by Formation of Large Aggregates with Diminished Noncovalent Interactions. Biomacromolecules.

[60] Yan S., Wang Q., Li Y., Qi B. (2024). Gallic acid-functionalized soy protein-based multiple cross-linked hydrogel: Mechanism analysis, physicochemical properties, and digestive characteristics. Food Chem..

